# Seizure-Associated ST Elevation Myocardial Infarction in Absence of Plaque Rupture

**DOI:** 10.1155/2018/6186521

**Published:** 2018-04-18

**Authors:** Haytham Mously, Mohammed Wazzan, Ahmed Z. Alkhathlan, Indiresha Iyer

**Affiliations:** ^1^Department of Internal Medicine, University Hospitals Cleveland Medical Center, Cleveland, OH, USA; ^2^Harrington Heart and Vascular Institute, University Hospitals Cleveland Medical Center, Cleveland, OH, USA

## Abstract

Acute coronary syndrome (ACS) is a very common cause of morbidity and mortality in the U.S. Here, we present a case of acute ST elevation myocardial infarction (STEMI) in the setting of seizure activity. In this rare case, we have data from optical coherence tomography (OCT) that showed no plaque disruption, showing the role of OCT in understanding the pathophysiology of STEMI and providing some ideas for the mechanism of this seizure-induced STEMI.

## 1. Introduction

Acute coronary syndrome (ACS) is a very common cause of morbidity and mortality in the U.S., accounting for approximately 1.5 million of hospital discharges and costing more than 150 billion dollars per year according to the American Heart Association [1]. Moreover, ACS includes unstable angina, non-ST elevation myocardial infarction (NSTEMI), and ST elevation myocardial infarction (STEMI). The pathophysiology includes atherosclerotic plaque rupture or plaque erosion [2]. NSTEMI occurs in the setting of subtotal occlusion of the culprit coronary artery. On the other hand, STEMI occurs due to complete occlusion of the culprit coronary artery [3]. In most cases, the sine qua non of acute myocardial injury is ST elevation on the 12-lead electrocardiogram (ECG). Other noncardiac conditions including acute cerebrovascular events can produce acute ST elevation but are rare [4, 5]. We present a case of acute ST elevation MI in the setting of seizure activity. In this rare case, we have data from optical coherence tomography that showed no plaque disruption.

## 2. Case Presentation

**Figure 1:**
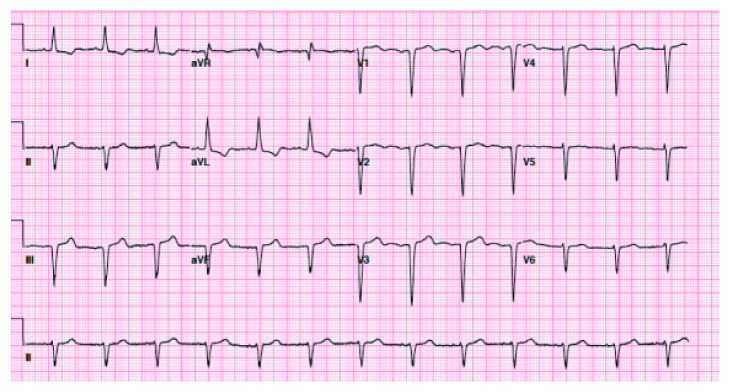
Baseline ECG upon admission showing inferior Q waves, nonspecific ST and T wave changes, and evidence of LVH.

**Figure 2:**
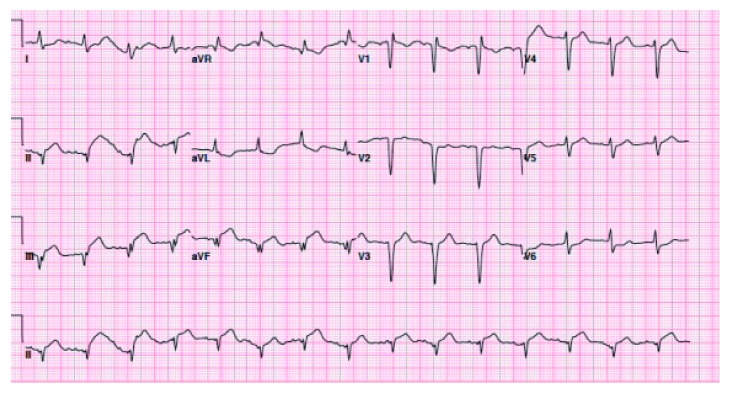
ECG after seizure showing acute interval development of ST elevations at leads II, III, and aVF.

**Figure 3:**
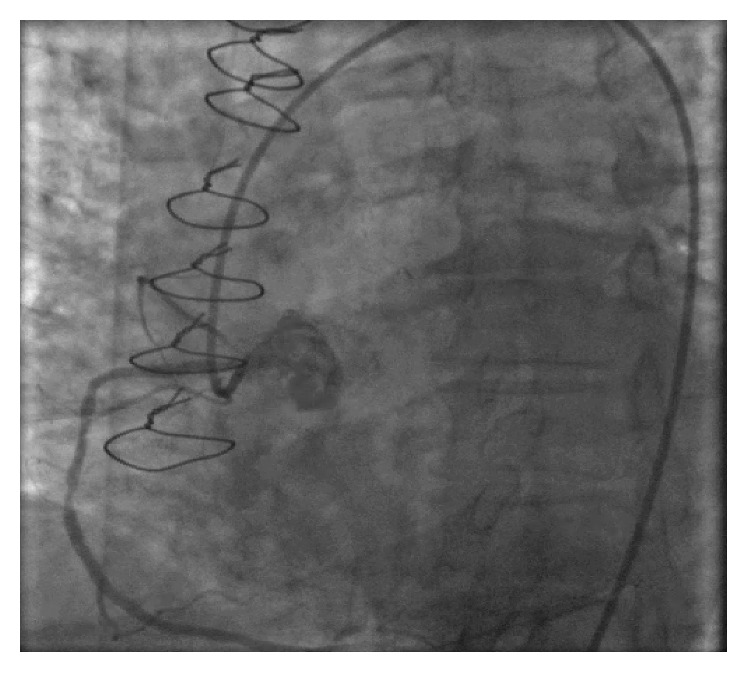
Mid-right coronary artery lesion.

**Figure 4:**
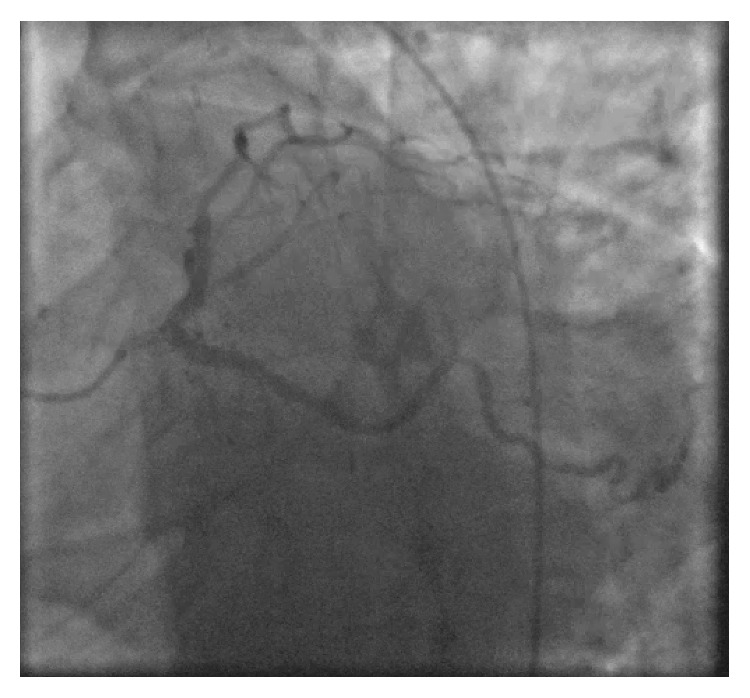
Left system angiography.

**Figure 5:**
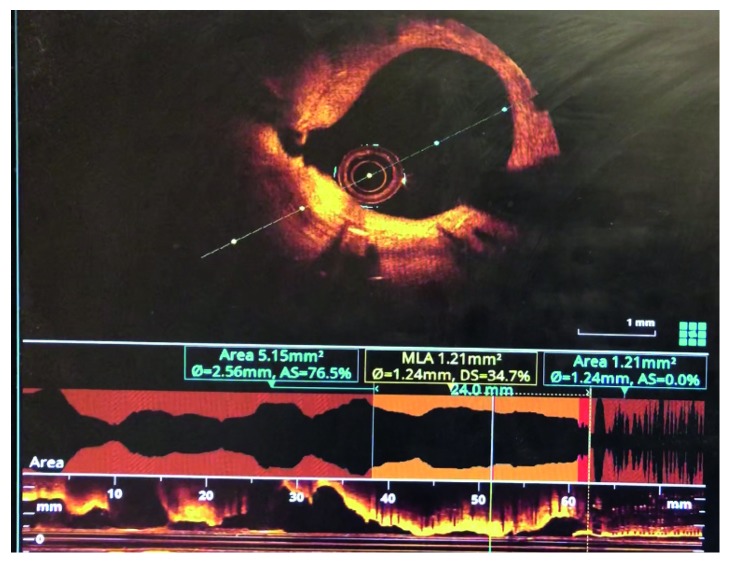
Optical coherence tomography.

**Figure 6:**
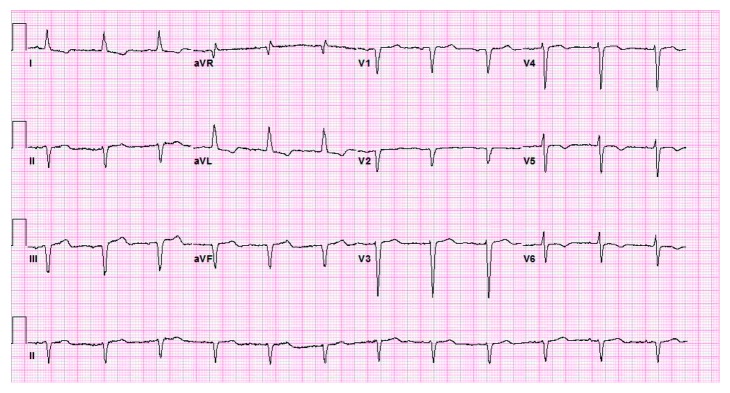
ECG after PCI showing marked resolution of ST elevation in leads II, III, and aVF.

A 75-year-old African American lady was admitted to the University Hospitals Richmond Medical Center for severe progressive headaches in the setting of hypertensive emergency. Her past medical history included coronary artery bypass graft (CABG) 20 years ago and subsequent multiple prior percutaneous coronary interventions (PCIs) to her right coronary artery (RCA) and left circumflex artery (LCx), and most recently 10 months before with a 2.75 × 14 mm Resolute Integrity drug-eluting stent (DES) for an in-stent restenosis of her mid-RCA. Echocardiogram obtained in 2015 revealed reduced ejection fraction of 40–45% and regional wall motion abnormalities involving apical septal and basal inferior segments and basal and mid inferior septum. She did not have any cardiac symptoms at the time of this admission. Her initial ECG showed sinus rhythm, left ventricular hypertrophy (LVH) by Cornell criteria, and inferior Q waves (Figure 1). Troponin I was 0.04 ng/mL (normal < 0.05 ng/mL). A noncontrast head CT showed no acute changes. She was admitted for further management. Hydralazine was used to gradually lower her elevated blood pressure. A routine second ECG 13 hours later at 04:53 AM showed similar findings. Serial troponin I values were 0.05 ng/mL and 0.05 ng/mL six hours apart. The next morning at 07:35 AM she suffered a tonic-clonic seizure that lasted for 5 minutes. She was treated successfully with lorazepam. An ECG at 08:03 AM after her seizure showed 2.5 to 3 mm acute ST elevations in leads II, III, and aVF (Figure 2). She was loaded with aspirin and ticagrelor and was started on heparin drip. Her blood pressure was 187/80 mmHg. A bedside limited echocardiogram showed EF 50% with hypokinesis in the basal and mid inferior walls and inferolateral walls. The 2.5 mm acute ST elevations in inferior leads were persistent for 40 minutes, and later she was transferred to the University Hospitals Cleveland Medical Center for primary PCI. Her coronary angiography showed a chronically occluded mid-LAD, a mid-LCx with a patent stent, and an 80% in-stent restenosis in the mid-RCA (RCA) at the site of prior intervention with TIMI flow 3 before intervention (Figures 3 and 4). Optical coherence tomography (OCT) (Figure 5) revealed malapposition of the prior overlapping stent and no thrombus or plaque rupture. She received a 2.5 × 38 mm Xience DES of her mid-RCA stenotic lesion, with good flow after intervention. Postprocedure ECG showed resolution of the ST segment elevation (Figure 6). The next day troponin I was 29.86 ng/ml and 16.76 ng/ml twelve hours apart. A complete transthoracic echo on day 3 after STEMI showed left ventricular ejection fraction of 50–55% and persistent basal and mid inferior wall and inferolateral wall hypokinesis. The patient's mental status remained altered and took two days to return to her baseline after PCI. The neurologist's main impression was posterior reversible encephalopathy syndrome (PRES) secondary to malignant hypertension causing seizures. She was discharged on beta-blockers, dual antiplatelet agents, amlodipine, and statin along with her other medications.

## 3. Discussion

Acute coronary syndrome is typically used to encompass a spectrum ranging from unstable angina, non-ST elevation myocardial infarction, and ST elevation myocardial infarction (STEMI) where the postulated mechanism involves plaque erosion or rupture which this patient did not have [1, 6–8]. This case shows that acute ST elevation on the ECG can occur without acute plaque erosion or rupture in the setting of seizure. ACh provocation was not performed to test for vasospasm. We postulate seizure-induced hypercatecholaminergic-mediated coronary vasospasm at the mid-RCA as the cause of the STEMI. Prior reports of acute ST elevation in noncardiac conditions such as subarachnoid hemorrhage and cocaine abuse postulate acute catecholamine surge causing cardiac myocyte and diffuse coronary vasospasm as the mechanism, especially at the sites of atherosclerosis [4, 9, 10]. The malapposition could have been identified and corrected if OCT had been utilized at the time of her prior intervention. Optical coherence tomography (Figure 5) is useful both to guide intervention and to understand pathophysiological mechanisms in patients with acute ST elevation on the ECG. ECG monitoring is important in patients with acute neurological conditions [11, 12].

## 4. Conclusions

Acute seizures can cause acute STEMI in the absence of plaque rupture or erosion. Optical coherence tomography is helpful to discern this type from others like plaque rupture or coronary artery dissection. Acute catecholamine surge–mediated vasospasm can be postulated as the cause of STEMI in this patient.

## References

[1] Kolansky D. M. (2009). Acute coronary syndromes: morbidity, mortality, and pharmacoeconomic burden. *American Journal of Managed Care*.

[2] Arbustini E., Dal Bello B., Morbini P. (1999). Plaque erosion is a major substrate for coronary thrombosis in acute myocardial infarction. *Heart*.

[3] Bhat P., Dretler A., Gdowski M., Ramgopal R., Williams E. D. (2016). *The Washington Manual of Medical Therapeutics*.

[4] Samuels M. A. (2007). The brain-heart connection. *Circulation*.

[5] Bailey W. B., Chaitman B. R. (2003). Electrocardiographic changes in intracranial hemorrhage mimicking myocardial infarction. *New England Journal of Medicine*.

[6] Kumar A., Cannon C. P. (2009). Acute coronary syndromes: diagnosis and management. *Part I. Mayo Clinic Proceedings*.

[7] Qiao J., Fishbein M. C. (1991). The severity of coronary atherosclerosis at sites of plaque rupture with occlusive thrombosis. *Journal of the American College of Cardiology*.

[8] Mann J., Davies M. J. (1999). Mechanisms of progression in native coronary artery disease: role of healed plaque disruption. *Heart*.

[9] Benedict C. R., Loach A. B. (1978). Sympathetic nervous system activity in patients with subarachnoid hemorrhage. *Stroke*.

[10] Schenk E. A., Moss A. J. (1966). Cardiovascular effects of sustained norepinephrine infusions. II. Morphology. *Circulation Research*.

[11] Osborn E. A., Jaffer F. A. (2013). Imaging atherosclerosis and risk of plaque rupture. *Current Atherosclerosis Reports*.

[12] Stamper D., Weissman N. J., Brezinski M. (2006). Plaque characterization with optical coherence tomography. *Journal of the American College of Cardiology*.

